# Experimental and Clinical Studies on the Effects of Natural Products on Noxious Agents-Induced Lung Disorders, a Review

**DOI:** 10.3389/fnut.2022.867914

**Published:** 2022-05-18

**Authors:** Saeideh Saadat, Sima Beigoli, Mohammad Reza Khazdair, Fatemeh Amin, Mohammad Hossein Boskabady

**Affiliations:** ^1^Applied Biomedical Research Center, Mashhad University of Medical Sciences, Mashhad, Iran; ^2^Department of Physiology, School of Medicine, Zahedan University of Medical Sciences, Zahedan, Iran; ^3^Cardiovascular Diseases Research Center, Birjand University of Medical Sciences, Birjand, Iran; ^4^Physiology-Pharmacology Research Center, Research Institute of Basic Medical Sciences, Rafsanjan University of Medical Sciences, Rafsanjan, Iran; ^5^Department of Physiology and Pharmacology, School of Medicine, Rafsanjan University of Medical Sciences, Rafsanjan, Iran; ^6^Department of Physiology, Faculty of Medicine, Mashhad University of Medical Sciences, Mashhad, Iran

**Keywords:** natural product, medicinal plants, bleomycin, cadmium, dust, lipopolysaccharide, sulfur mustard, lung injury

## Abstract

The harmful effects of various noxious agents (NA) are well-known and there are reports regarding the induction of various lung disorders due to exposure to these agents both in animal and human studies. In addition, various studies have shown the effects of natural products (NP) on NA-induced lung disorders. The effects of various NP, including medicinal plants and their derivatives, on lung injury induced by NA, were reviewed in this study. The improving effects of various NP including medicinal plants, such as *Aloe vera, Anemarrhena asphodeloides, Avena sativa, Crocus sativus, Curcuma longa, Dioscorea batatas, Glycyrrhiza glabra, Gentiana veitchiorum, Gentiopicroside, Houttuynia cordata, Hibiscus sabdariffa, Hochu-ekki-to, Hippophae rhamnoides, Juglans regia, Melanocarpa fruit juice, Mikania glomerata, Mikania laevigata, Moringa oleifera, Myrtus communis L., Lamiaceae, Myrtle, Mosla scabra leaves, Nectandra leucantha, Nigella sativa, Origanum vulgare L, Pulicaria petiolaris, Paulownia tomentosa, Pomegranate seed oil, Raphanus sativus L. var niger, Rosa canina, Schizonepeta tenuifolia, Thymus vulgaris, Taraxacum mongolicum, Tribulus Terrestris, Telfairia occidentalis, Taraxacum officinale, TADIOS, Xuebijing, Viola yedoensis, Zataria multiflora, Zingiber officinale, Yin-Chiao-San*, and their derivatives, on lung injury induced by NA were shown by their effects on lung inflammatory cells and mediators, oxidative stress markers, immune responses, and pathological changes in the experimental studies. Some clinical studies also showed the therapeutic effects of NP on respiratory symptoms, pulmonary function tests (PFT), and inflammatory markers. Therefore, the results of this study showed the possible therapeutic effects of various NP on NA-induced lung disorders by the amelioration of various features of lung injury. However, further clinical studies are needed to support the therapeutic effects of NP on NA-induced lung disorders for clinical practice purposes.

## Introduction

The respiratory function of the lung is critical and important for survival because oxygen is a vital molecule for the production of energy that is essential for the life of organisms (1). Studies have shown the effects of exposure to air pollutants on respiratory disorders such as COPD, asthma, and lung cancer (2). Specific alterations of passive and active non-respiratory functions generate functional or anatomical disorders that compromise breathing later. The basic scientific and clinical research of various diseases generated by alterations of these functions can produce knowledge on the pathophysiology, biochemistry, genetics and immunology (3).

Respiratory disorders such as COPD and asthma are related to immune and inflammatory reactions and the status of oxidants which were remarkably enhanced in respiratory disorders (4). Allergic disorders have increased in recent decades due to increased allergens and air pollutants in the environment and workplace (5). In allergic disorders such as skin and respiratory allergies, the reaction of the immune system to exposure and re-exposure to allergens releases allergy-related mediators (6).

Bleomycin (BLM) is a type of antibiotic used for cancer chemotherapy. This drug reduces or stops the growth of cancer cells in the body. It inhibits DNA metabolism and is used as an antineoplastic (anticancer) agent, especially for solid tumors. At high concentrations of the drug, protein production and cellular RNA are also inhibited. It has the least toxic effect on blood-forming tissues and the immune system. Unfortunately, due to the complication of pulmonary fibrosis (PF), the use of this drug is clinically limited (7).

Cadmium (Cd) is a naturally occurring toxic element. Several studies have shown that exposure to Cd from cigarette smoking and occupational resources causes lung disorders. There are reports regarding the involvement of pro-inflammatory chemokines and cytokines, such as interleukins, growth factors, and nuclear factor kappa B (NF-κB), a transcription factor that regulates the expression of genes of cytokines which play an important role in pulmonary fibrosis due to Cd exposure (8, 9).

Studies have shown that dust particles may penetrate deep into the lungs, throat, and airways and cause respiratory disorders. The entry of dust into the lung parenchymal macrophage cells leads to the chemical secretion of chemotaxis and inflammatory mediators, leukotrienes and thromboxane, causing the invasion of inflammatory cells from the vessels to the lung damage area. This process, in turn, stimulates the synthesis of the fibroblasts and causes fibrotic pulmonary parenchyma (10). It was also reported that lipopolysaccharide (LPS) causes lung damage (11) through several inflammatory mechanisms (12).

Paraquat (PQ) causes human or animal toxicity and the lungs are the primitive target organ due to being the main exposed organ to this toxin (13). The effects of PQ on the lung result in lung edema, hypoxia, and lung fibrosis (14, 15). Also, the effects of PQ on interleukin 6 (IL 6) and tumor necrosis factor-α (TNF α) in the macrophages have been reported (16, 17). It was also shown that the mechanisms of pulmonary injuries caused by the PQ are mainly related to the inflammatory and oxidative stress processes (18). Chemical agents such as sulfur mustard (SM) might cause acute and chronic injuries in the lung tissue (19) due to enhanced inflammatory oxidant stress mechanisms (20, 21).

Medicinal herbs are applied for the medical treatment of various disorders (22). People use different products from plant resources traditionally for the treatment of respiratory disorders including asthma and bronchitis (22). Several natural ingredients such as polyphenols, flavonoids, and alkaloids derived from medicinal plants showed potent anticancer activity (23).

Natural products (NP) can be considered as the alternative therapeutic potential for respiratory diseases caused by toxic agents since different inflammatory mediators are involved in these disorders and several NP showed anti-inflammatory effects. Most of the studies are pointing out the effects of NP on the inhibition of NF-κB and MAPK pathways, besides the antioxidant effects associated with these products. However, clinical trials using these compounds are scarce in the literature and the safety and efficacy should be confirmed for further studies.

Since no study has been done on the effect of NP on the noxious agents-induced lung disorders so far, this review article is to present available basic and clinical evidence about the efficacy of the mentioned NP and the herbal constituents in the prevention or treatment of lung disorders induced by the noxious agents-induced similar inflammatory and pathological changes in the lung as induced by BLM, Cd, dust, and LPS in experimental and SM in clinical models. Therefore, the effects of NP and their constituents on noxious agents-induced lung changes were also suggested in the present review to support their effect on lung changes induced by noxious agents in clinical studies.

## Method

In this review, the keywords including “chemical agent” and “medicinal plants” or “natural products” and “lung injury” or “respiratory system” were searched on different databases such as Web of Science, PubMed, and Scopus from 1989 to the end of September 2021.

**Figure 1:**
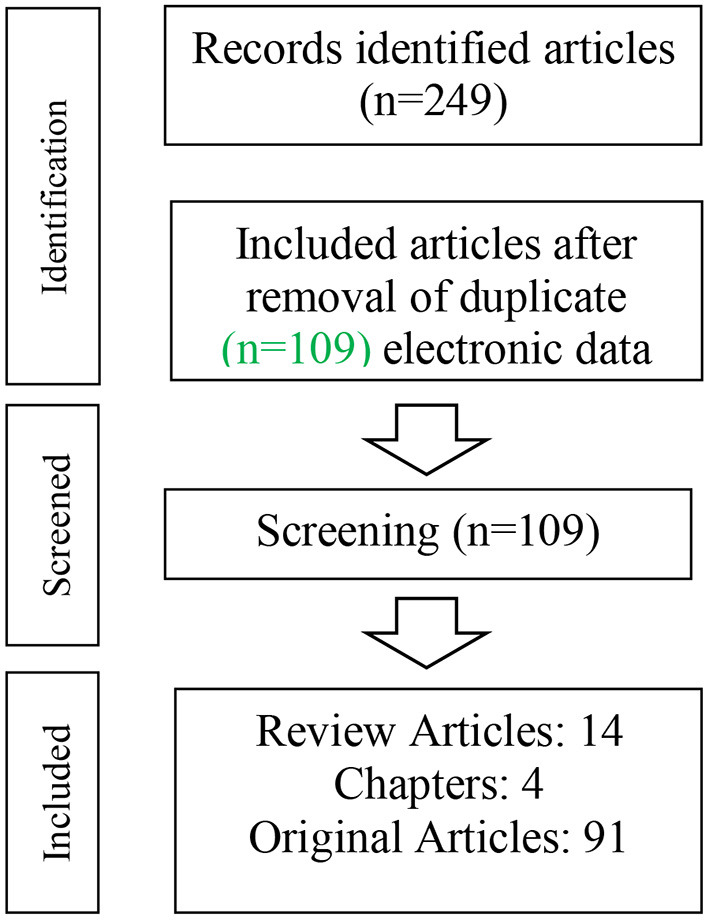
Flowchart of the process of selecting studies for review.

In total, 224 articles were retrieved including 115 duplicates articles. Therefore, 109 articles (14 reviews, 4 book chapters, and 91 original articles) related to the described topic were included in this review article (Figure 1).

## Results

### Bleomycin-Induced Lung Disorders

#### Experimental Studies

The prophylactic effect of common walnut *(Juglans regia)* (150 mg/kg) on rats exposed to BLM was shown by increased glutathione reductase (GR) and catalase (CAT) levels and decreased lung inflammation and apoptosis through regulation of NF-κB activity. The treatment with *Juglans regia* also causes modulated lung injury through markers of cellular injury including lactate dehydrogenase (LDH), alkaline phosphatase, and reduced glutathione (GSH) (24).

*Gentiana veitchiorum* treatment decreased collagen VI and improved the lung injury induced by BLM. The treatment with *Gentiana veitchiorum* also decreased the malondialdehyde (MDA) level and increased the superoxide dismutase (SOD) and GSH activities, which correlated with oxidation resistance and scavenging of free radicals. Finally, *Gentiana veitchiorum* deceased the inflammatory lung damages through the alleviation of tumor necrosis factor-alpha (TNF-α) expression (25).

In the BLM-induced lung inflammation and pulmonary fibrosis (PF) mouse model, treatment with *Juglanin* (80 mg/kg), that was mainly divided from the green walnut husks of *Juglans mandshuric*, improved the survival rate in the treated mice. Also, the PF induced by the BLM was markedly attenuated by *Juglanin* with the decreasing of the expression of the transforming growth factor-β1 (TGF-β1), metallo-proteinase-9 (MMP-9), α-smooth muscle actin (α-SMA), and collagen I (26).

The treatment with a Japanese herbal medicine, Hochu-ekki-to (TJ-41), in a mouse model of BLM-induced PF, for 2 months before and 1 month after receiving BLM, prevented PF through the modification of the Th_1_/Th_2_ imbalance toward the Th_2_ balance (27). *Feitai* is a Chinese herb used for the treatment of systemic inflammatory response syndrome (SIRS) and multiple organ dysfunction syndrome (MODS). In the BLM-induced pulmonary fibrosis in rats, *feitai* blocked lung p38 MAPK, NF-κB65, HIF-1α, p-IκB-α, and TGF-β1 expression, and enhanced the Nrf2 and IkB expression (28).

The administration of *Rosmarinus officinalis* L. extract (75 mg/kg) protected against the BLM-induced acute lung injury in the animal model *via* declines in lung edema, septal thickening, alveolar subsidens, hemorrhage, and oxidative stress (29). The findings of the other study showed that apigenin (4, 5, 7-trihydroxyflavone) with doses 10, 15, and 20 mg/kg is a potent anti-inflammatory and antifibrotic agent against the BLM-induced PF (30).

The effects of indirubin, a compound derived from mollusks of the family Muricidae, on the BLM-induced PF were examined by pathological staining, western blot, RT-PCR, and immunofluorescent staining. The treatment with indirubin protected the mice against the BLM-induced PF by alleviated fibroblast differentiation indicating its possible therapeutic effect on PF (31). In a model of PF induced by a single endotracheal injection of BLM, the extract of *Nigella sativa* (500 mg/kg) was effective for early and late prevention of PF and inflammation (32).

The administration of *Raphanus sativus L. var niger* (RSN), from the black radish plant, ameliorated the BLM-induced acute lung injury. The post-treatment of rats with intravenously administered RSN (75, 150, 300 mg/kg) protected the lung against the BLM-induced oxidative stress and reduced the number of neutrophils and lymphocytes as well as the IL-6, TNF-α, IL-1β, and TGF-β1 levels (33). *Houttuynia cordata* (HC) has shown antioxidant activity, free radical scavenging capacity as well as anti-inflammatory and anticancer activities. These results suggested that *Houttuynia cordata* has a protective effect against BLM-induced PF (34).

The *Myrtus communis* L. extract (50 mg/kg) effectively inhibited the inflammation and fibrosis of lung parenchyma in a rat model of BLM-induced pulmonary injuries. This impact might be due to the decrement of tissue inflammation and inhibition of oxidative stress (35). The treatment with resveratrol (10 mg/kg), a phenolic compound, prevented the BLM-induced PF in the rats by the suppression of oxidative stress and endothelin-1 (ET-1) expression. The results demonstrated that resveratrol with its potent free radical scavenging and antioxidant properties seems to be a highly promising agent in protecting lung tissue against oxidative damage and in preventing PF due to BLM treatment (36).

Ganoderic acid A has been shown to mitigate the increment in NF-κB p65, TNF-α, and IL-1β and IL-6 mRNA expression, and improved the expression of the anti-inflammatory cytokine IL-10 following the BLM injection. The treatment with ganoderic acid A (25 and 50 mg/kg, for 3 weeks) significantly improved the MPO activity and lung histopathology in the mice. Also, the protective effect of ganoderic acid A may be related to a decrease in TNF-α, IL-1β, IL-6, and MDA and an increment of SOD (37). The gallic acid (75, 150, and 300 mg/kg, for 3 weeks) in an animal model of BLM-induced PF reduced the inflammation process to some extent and could exert its effects through TGF β1/Smad2-signaling pathway and balancing NOX4/factor erythroid-2-related factor 2 (Nrf2) (38). Yin-Chiao-San (YCS), a Kampo medicine, is widely applied for pulmonary diseases. The treatment of rats with YCS (1,000 mg/kg/day, i.v.) protected the lung against the BLM-induced and reduced the lung index, MDA, HP, and TNF-α as well as significantly enhanced the CAT activity (39).

**Figure 2:**
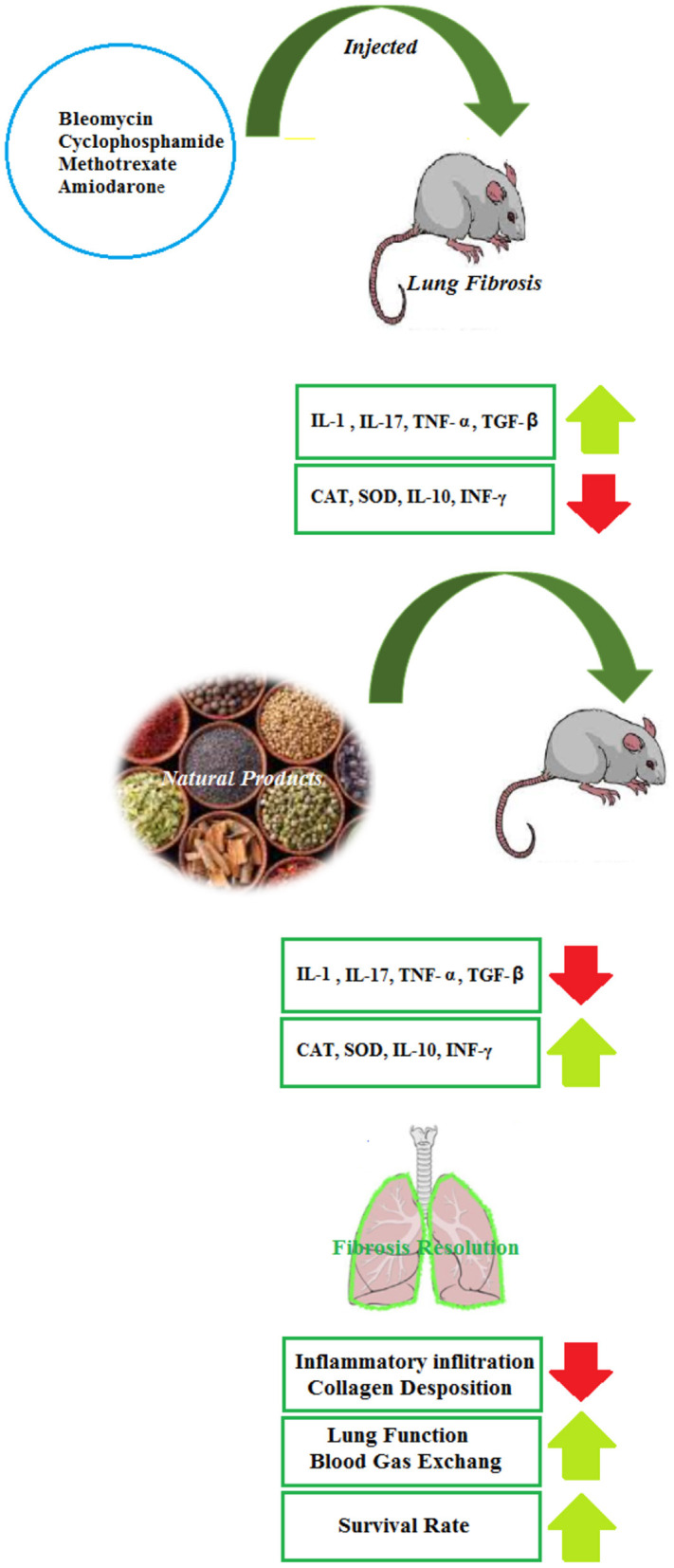
Inhibitory effects of NP on pulmonary fibrosis induced by various noxious agents.

Several reports have evaluated the effects of NP on the BLB-induced lung disorders in experimental models and it is suggested that the herbs and their active ingredients are a promising source of compounds that can play pivotal roles in the alternative adjuvant chemotherapy in reducing the pulmonary fibrosis of BLM. However, clinical trials in this field are not found and should be performed in the future. The therapeutic effects of NP in the BLM-induced lung injury are summarized in Table 1 and Figure 2.

**Table 1:** The possible therapeutic effects of NP in the BLM-induced lung injury.

**Study type**	**Study design**	**NP**	**Dose**	**Effects**	**References**
*In vivo*	BLM-exposed rats	*Juglans regia*	150 mg/kg, for 14 days	↑ GR and CAT activities ↓ LDH, ALP, GSH Apoptosis via regulate the NF-κB signaling pathway	(24)
	BLM-exposed rats	*Gentiana veitchiorum*	-	↓ Inflammatory lung injury by decreasing TNF-α expression and MDA ↑ SOD, GSH	(25)
	BLM-exposed mice	Juglanin	80 mg/kg, i.p. for 4 weeks	↓ Expression of TGF-β1, MMP-9, α-SMA, collagen I	(26)
	BLM-exposed mice	TJ-41	1g/kg, orally for 13 weeks	Prevented experimental lung fibrosis through the correction of the Th_1_/Th_2_ imbalance	(27)
	BLM-exposed rats	*Feitai*	-	↓ Oxidative stress and lung inflammation	(28)
	BLM-exposed rats	*Rosmarinus*	75 mg/kg, i.p. 15 days	↓ Lung edema, septal thickening, alveolar subsidence, hemorrhage and oxidative stress	(29)
	BLM-exposed rats	Apigenin	10, 15 and 20 mg/kg, orally for 14 days	↑ CAT, SOD activities, IL-10 and INF-γ	(30)
	BLM-exposed rats	Indirubin	12.5 mg/kg, or 25 mg/kg, i.p. for 14 days	Alleviated fibroblast differentiation	(31)
	BLM-exposed rats	*Nigella sativa*	500 mg/kg, i.p. for 14 days	Prevented pulmonary fibrosis and inflammation	(32)
	BLM-exposed rats	RSN	150 mg/kg, orally for 7 days	↓ TGF-β1 level	(33)
	BLM-exposed mice	HC	50 and 100 mg/kg, i.g. for 5 weeks	↓ Oxidative damage	(34)
	BLM-exposed mice	Resveratrol	10 mg/kg, orally for 14 days	↓ Oxidative damage Prevented pulmonary fibrosis	(36)
	BLM-exposed rats	*Myrtus communis*	50 mg/kg, i.g for 14 days	↓ Tissue inflammation Inhibition of oxidative stress	(35)
	BLM-exposed rats	Gallic acid	50, 100 and 200 mg/kg, orally for 14 days	↓ Serum levels of IL-4, IL-17A, IFN γ	(40)
	BLM-exposed rats	GAA	25 and 50 mg/kg, i.g. for 21 days	↑ NF-κB, TNF-α, IL-1β and IL-6	(37)
	BLM-exposed rats	Gallic acid	75, 150 300 mg/kg, i.g. for 21 days	↑ CAT, SOD activities, IL-10 and INF-γ	(38)
	BLM-exposed mice	YCS	1,000 mg/kg for 5 days i.p.	Antioxidant and anti-inflammatory activities and also inhibited collagen formation	(39)

*NP, natural products; Ext, extract; BLM, bleomycin; TGF-β1, transforming growth factor-β1; MMP-9, metallo-proteinase-9; α-SMA, α-smooth muscle actin; TNF- α, tumor necrosis factor alpha; IFNγ, Interferon gamma; IL-10, Interleukin-10; IL-17, Interleukin-17; SOD, superoxide dismutase; CAT, catalase; RSN, Raphanus sativus L. var niger; HC, Houttuynia cordata; TJ-41, hochu-ekki-to; GR, glutathione reductase; GSH, glutathione; LDH, lactate dehydrogenase; ALP, alkaline phosphatase; GAA, Ganoderic acid A; i.g., intragastrically; YCS, Yin-Chiao-San*.

*The up arrow (↑) indicates an increase in the variable, and a down arrow (↓) indicates a decrease*.

### Cadmium-Induced Lung Disorders

#### Experimental Studies

**Figure 3:**
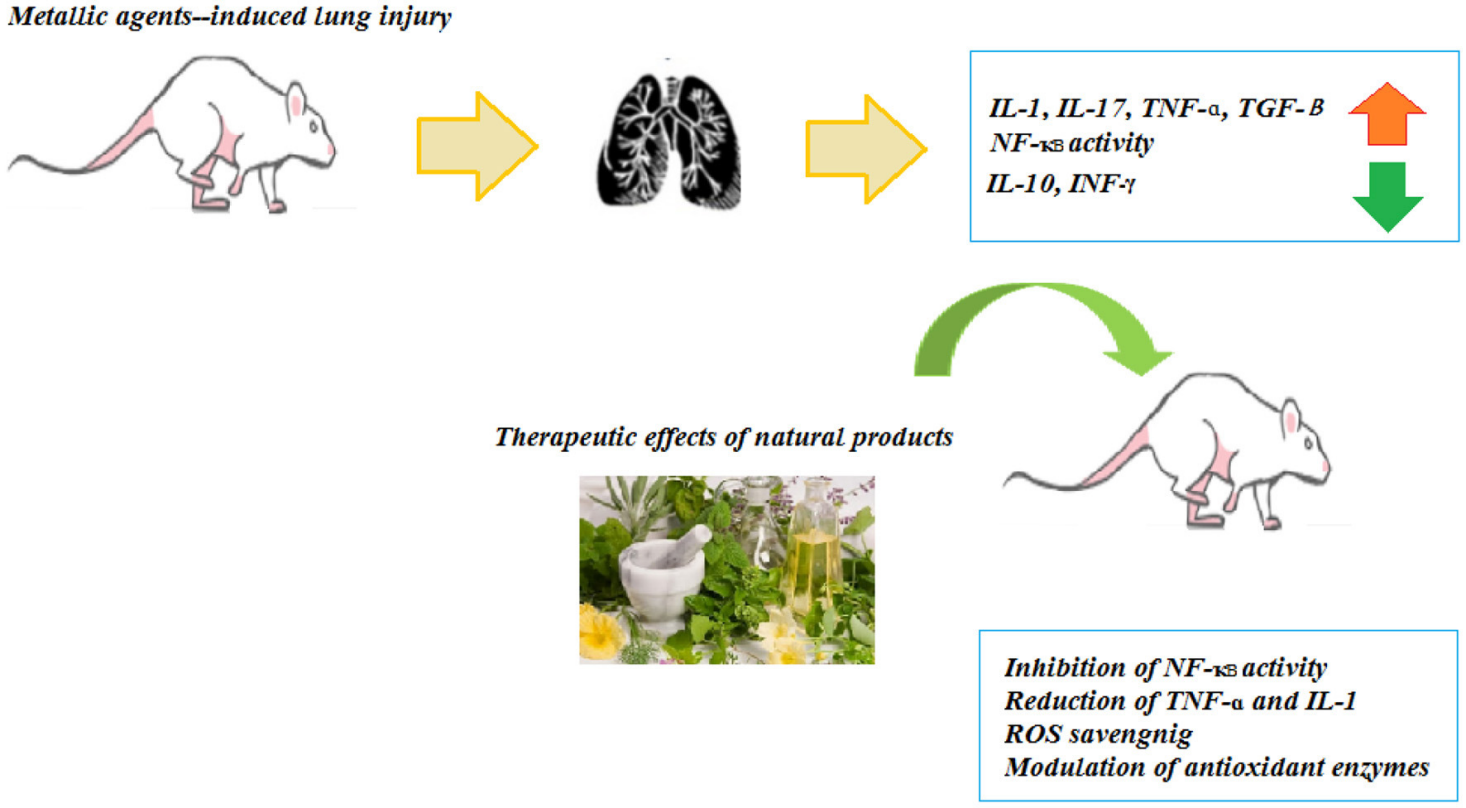
Inhibitory effects of NP on metallic agents-induced pulmonary fibrosis.

The administration of *Nigella sativa* oil (1 ml/kg, i.p.) ameliorated the Cd-induced lung damage with the reduction of histopathological changes in the lung architecture (41). The treatment effects of *Tribulus Terrestris* against the Cd-induced toxicity in the mice showed that the alcoholic extract of *Tribulus Terrestris* fruit (200 mg/kg, for 10 days) eliminated the free radicals and increased the antioxidant enzymes expression as well as the down-regulation of pro-inflammatory markers in cellular injuries (42). The anti-inflammatory effects of the phenolic compounds from grape seeds were associated with their regulatory effect on the expression of the pro-inflammatory genes, such as cyclooxygenase and lipoxygenase and also by acting on the NF-κB signaling and MAPK. The findings showed that the phenolic compounds of the grape seeds ameliorate the toxic impacts of Cd in the lung tissue *via* its free radical scavenging property, antioxidant activity, and antiapoptotic potential (43). In the rabbits challenged with Cd (6 mg/kg, i.p.) and treated with pomegranate seed oil (0.8 ml/kg), a significant decrease in the blood volume and hemoglobin was seen (44). Some herbs and NP exhibited significant protection on Cd-induced respiratory insults in experimental animal models and pre-clinical studies but the clinical studies were not found in this regard. The findings from these studies may lead to new therapeutic development for a new drug for the treatment of Cd-induced respiratory injuries. These studies may also guide other investigators to develop quality NP clinical trials in the future. The effects of NP on Cd-induced lung injury are summarized in Table 2 and Figure 3.

**Table 2:** The possible therapeutic effects of NP in the Cd-induced lung injury.

**Study type**	**Study design**	**NP**	**Dose**	**Effects**	**References**
*In vivo*	Cd-exposed mice	*Nigella sativa*	1 ml/kg, i.p. for 28 days	Ameliorated Cd-induced lung damage with minimal histopathological changes in lung architecture	(41)
	Cd-exposed mice	*Tribulus Terrestris*	200 mg/kg, i.p. for 10 days	Eliminate free radicals ↓ Antioxidant enzymes expression, down-regulated proinflammatory markers in cellular injuries	(42)
	Cd-exposed mice	Grape seeds Ext	-	Improved hazard toxic effect on the lung tissue, antioxidant activity and anti-apoptotic potential	(43)
	Cd-exposed rabbits	Pomegranate seeds oil	0.8 ml/kg, for 30 days	↓ Blood volume, hemoglobin, and improved lung function	(44)

*NP, natural products; Ext, extract; Cd, Cadmium; i.p., intraperitoneal*.

*The up arrow (↑) indicates an increase in the variable, and a down arrow (↓) indicates a decrease*.

### Environmental Dust-Induced Lung Disorders

#### Experimental Studies

Pneumoconiosis is a lung disease caused by certain kinds of dust particles in the workplace. In rats exposed to an inoculation inside the trachea, coal dust (3 mg/0.3 ml of saline), treatment with *Mikania glomerata* and *Mikania laevigata* extracts (100 mg/kg, s.c., for 2 weeks) prevented the increase in the total cell count and LDH activity in the bronchoalveolar lavage fluid (BALF), and diminished the lung inflammatory infiltration induced by the coal dust, as assessed by the histopathologic analyses. These findings suggested that both extracts showed a protective effect on the oxidation of thiol groups (45).

In rats exposed to uranium ore dust inhalation (period of 3, 7, 30, and 60 days), the administration of licorice (*Glycyrrhiza glabra*) aqueous extract normalized the pyruvic acid contents in the lung tissue during the study periods and decreased the lactic/pyruvic acid ratio (46).

In albino rats exposed to cement dust, the antioxidant activities of roselle (*Hibiscus sabdariffa*), moringa (*Moringa oleifera*), ginger (*Zingiber officinale*), and ‘ugwu' (*Telfairia occidentalis*), fed with herbal extracts (400 mg/kg) for 6 months were evaluated. The lungs of non-treated rats showed severe interstitial fibrosis and cellular debris. Moderate fibrosis was seen in the lung tissues of the rats treated with *Hibiscus sabdariffa* and *Moringa oleifera* extracts. The rats that were fed with the mixture of the extracts had mild septal fibrosis (47).

Treatment with *Hibiscus sabdariffa, Moringa oleifera, Zingiber officinale*, and *Telfairia occidentalis* in the rats exposed to cement dust showed moderate to normal biochemical parameters when compared with the non-treated rats. Higher hematological parameters were observed in the treated rats than in the non-treated rats. Overall, the mixture of extracts decreased the adverse effects of cement-dust exposure more than any individual extract alone. Individually, *Telfairia occidentalis* performed the best, followed by *Zingiber officinale, Moringa oleifera*, and *Hibiscus sabdariffa* (48). The elemental analysis of the cement dust shows that it contains 57% calcium, 23% silicon, 10.5% aluminum, 8.5 % chromium, and 8.0 % lead (47). The data of these studies indicated the antioxidant properties of the food plants which modulated the effects of cement dust. Hence, the plants could be used as supportive care in polluted environments to lower the health problems associated with cement-dust exposures. These experimental studies indicated that *Hibiscus sabdariffa, Moringa oleifera, Zingiber officinale, Telfairia occidentalis, Mikania glomerata, Mikania laevigata*, and *Glycyrrhiza glabra* might be candidates for the prevention of lung injury caused by swine barn, coal, uranium ore, and cement-dust exposure. These results suggested a potential role for oxidative stress pathways in mediating occupational lung diseases and antioxidants effect of the plants in reducing dust-mediated oxidative stress in lung disorders of exposed workers.

This review study indicates the therapeutic effect of NP on dust-induced lung disorders but more clinical studies are required to establish the clinical efficacy of these plants and their constituents on lung and allergic disorders. The therapeutic effects of NP including anti-inflammatory effects and reduction of airway responsiveness in the animal models of asthma and COPD were also illustrated. Different NP and their components were identified as anti-asthmatic components. We suggest the possible therapeutic effect of NP on lung disorders of dust-exposed patients *via* the decrement of inflammatory and enhanced anti-inflammatory mediators, and improved pulmonary function tests. The effects of NP on dust-induced lung disorders are summarized in Table 3.

**Table 3:** The possible therapeutic effects of medicinal herbs in the dust-induced lung disorders in experimental studies.

**Study type**	**Study design**	**NP**	**Dose**	**Effects**	**References**
*In vivo*	Coal dust-exposed rats	MGE and MLE Exts	100 mg/kg, for each Ext, s.c., for 2 weeks	↓ Total cell count and LDH activity ↑ Protein sulfhydryl content in lung	(45)
	Uranium ore dust-exposed rats	*Glycyrrhiza glabra* aqueous Ext	100 mg/kg	Normalized pyruvic acids cand actic/pyruvic acids ratio in lung tissue	(46)
	Cement dust-exposed rats	*Hibiscus sabdariffa, Moringa oleifera, Zingiber officinale* and *Telfairia occidentalis* ethanolic Ext	400 mg/kg, orally, for 180 days ratio 1:1:1:1	Decrease in lung fibrosis	(47)
	Cement dust-exposed rats	*Hibiscus sabdariffa, Moringa oleifera, Zingiber officinale* and *Telfairia occidentalis* ethanolic Ext	400 mg/kg, orally, for 180 days (100 mg of each Ext)	↓ Serum protein, ALT, AST and ALP and lung histological changes ↓ Toxic elements accumulation in the lung	(48)

*NP, natural products; Ext, extract; ALP, alkaline phosphates; ALT, alanine amino transferase; AST, aspartate amino transferase; MGE, Mikania glomerata; MLE, Mikania laevigata*.

*The up arrow (↑) indicates an increase in the variable, and a down arrow (↓) indicates a decrease*.

### Lipopolysaccharide-Induced Lung Disorders

#### Experimental Studies

The administration of the extract of *Paulownia tomentosa* stem bark (2.5, 5, 10, 20, and 40 μg/ml, for 24 h), represses the release of IL-6 and TNF-α in the RAW 264.7 macrophages stimulated by LPS (49). In this cell line, dehydrodieugenol B from *Nectandra leucantha* (10, 20, 30, and 60 μM) did not influence the cell viability but hindered the enhancement in IL-1β and IL-6 gene expression and NO release (50).

*In vitro*, Barbaloin (a major anthraquinone compound) (25, 50, or 100 μM), decreased the expression of TNF-α, IL-1β, and IL-6 as well as the activation of ROS-mediated PI3K/AKT/NF-κB pathway dose-dependently (51). The ethanolic extract of the aerial parts of *Houttuynia cordata* (30, 50, 100, and 300 μg/mL) also inhibited the iNOS-mediated NO release from the LPS-stimulated MH-S cells (a mouse alveolar macrophage cell line) concentration-dependently (52).

Incubating the RAW264.7 cells with LPS and alpinumisoflavone (1, 5, and 10 μg/mL, for 24 h), a plant-derived pyranoisoflavone remarkably inhibited the release of NO, cytokines, and ICAM-1 protein expression. Treatment with alpinumisoflavone blocked the IκBα phosphorylation and degradation and decreased the phosphorylation of IKK and NF-κB. In addition, it effectively decreased the phosphorylation of ERK, Jc-Jun-NH2 terminal kinase (JNK), and p38. In LPS activation of the NLRP3 inflammasome, caspase-1, and IL-1β proteins were inhibited by alpinumisoflavone, especially at its high dose. The alpinumisoflavone treatment also remarkably decreased the IL-17A and iNOS protein expression but it did not block the LPS-induced cyclooxygenase-2 (COX-2) induction. Furthermore, alpinumisoflavone significantly enhanced the expression of antioxidant enzymes dose-dependently. The LPS induction of intracellular ROS production was also significantly inhibited by the treatment with alpinumisoflavone (53).

The treatment of LPS-stimulated and non-stimulated splenocytes with the aqueous extract of *Curcuma longa* (0.8–500 μg/mL, for 48 h), remarkably enhanced NO, pro-inflammatory cytokines, tumor necrosis factor, interferon-gamma, and monocyte chemoattractant protein-1(MCP-1). The levels of IL-12 and PGE2 in the LPS-stimulated cells were also inhibited by the plant extract (54). In the LPS-activated epithelial cell line, the levels of NF-κB p52, NF-κB p65 transcription factors protein, IL-1ß, interleukin-8 (IL-8), and mucus secretion were significantly reduced by the hydroalcoholic extract of *Thymus vulgaris* (0.04–0.60%) (55).

Daidzein (100 μM,), a diphenolic isoflavone 15 min after LPS stimulation, obviously inhibited the expressions of myeloid differentiation factor 88 (MyD88), toll-like receptor 4 (TLR4), and the activation of NF-κB in the A549 alveolar epithelial cells stimulated by 10 μg/ml LPS (56). In the RAW264.7 cells, treatment with a mixture of *Taraxacum officinale* (a herbal formulation) (0.5, 1, and 2 mg/mL), repressed the LPS (100 ng/mL)-induced inflammatory responses (57). Also, the expression of pro-inflammatory cytokines activated the Nrf2-HO-1 axis and oxidative stress was inhibited in the treated LPS-stimulated cells (57).

Eugenol and dehydrodieugenol B from *Nectandra leucantha* (30 mg/kg) in the mice with the LPS-induced ALI, decreased lung edema, inflammatory cells, and the IL-6 and IL-1 β levels in the BALF as well as decreased inflammatory cell infiltration and those positive to iNOS, MMP-9, and TIMP-1, and decreased the collagen content and the 8-isoprostane expression in the lung tissue (50).

In LPS-challenged mice, treatment with thymol (30 and 100 mg/kg, i.p.), one of the primary active constituents derived from *Thymus vulgaris*, before or after the LPS challenge, significantly improved the pathological changes in the lung tissues. Thymol also inhibited the LPS-induced inflammatory cells influx and protein concentration in the BALF. Additionally, thymol markedly inhibited the LPS-induced elevation of MDA and MPO levels as well as reduction of the SOD activity. Thymol also effectively inhibited the NF-κB activation in the lung (58). In the LPS-induced ALI, treatment with methanolic extract of *Pulicaria petiolaris* (50 and 100 mg/kg, p.o., for 5 days) reduced pulmonary edema, ameliorated the LDH level in the BALF, improved the histopathological lesions in the lung tissue, and showed antioxidant capacity (59).

The administration of a single dose of cannabidiol (0.3, 1.0, 10, 20, 30, 40, and 80 mg/kg, i.p.), extracted from *Cannabis sativa*, before the LPS-induced ALI, decreased the migration of leukocytes into the lungs, albumin concentration in the BALF, production of pro-inflammatory cytokines and chemokines, and MPO activity in the lung tissue. In addition, in the LPS-induced ALI, ZM241385 (a selective adenosine A2A receptor antagonist), inhibited all anti-inflammatory effects of cannabidiol which indicate the contribution of adenosine A2A receptor in the anti-inflammatory effects of cannabidiol (60).

The linalool (25 mg/kg, i.p.) treatment attenuated the production of LPS—decreased the changes in TNF-α and IL-6 as well as lung histopathologic changes in the ALI mouse model (61). In the mice with the LPS-induced ALI, Barbaloin extracted from *Aloe vera* ameliorated lung pathological changes such as infiltration of inflammatory cells, alveolar hyperemia, necrosis, and lung epithelial cell detachment (51).

The treatment with the aqueous extract of *Taraxacum mongolicum* Hand.-Mazz (5 and 10 g/kg, p.o.) inhibited the LPS-induced lung injury in female BALB/c mice by reducing the inflammatory cell infiltration in the BALF, lung protein levels and PI3K/Akt/mTOR signaling. It also improved the activity of SOD and inhibited the MPO activity (62).

The *Portulaca oleracea* extract (50, 100, and 200 mg/kg, p.o., 1 h before LPS injection) suppressed the LPS-induced rat ALI by decreasing IL-6, IL-β, TNF-α, TGF-β, and, PGE2 but increasing IL-10 levels. *Portulaca oleracea* improved the levels of the white blood cells (WBC), MDA, MPO, and thiol as well as SOD and CAT activities. The lung wet/dry ratio (an index of interstitial edema) was also significantly reduced. Therefore, the *Portulaca oleracea* extract displayed antioxidant and anti-inflammatory activity dose-dependently on the LPS-induced ALI model in the rat model (63).

In the LPS-induced ALI, xanthohumol (10 or 50 mg/kg, i.p.), a prenylflavonoid extracted from the hop plants (*Humulus lupulus*) (0, 18, 35, and 70 μmol/kg, i.p., for 30 min), showed a protective effect against oxidative stress and inflammatory damage by regulation of the Nrf2 pathway through AMPK/GSK3β activation, and suppression of LPS-activated Txnip / NLRP3 inflammation and the NF-κB signaling pathway (64). In LPS-induced lung inflammation in mice, pre-treatment with luteolin (0, 18, 35, and 70 μmol/kg, i.p., for 30 min) decreased IL-6 and TNF-α levels and expression of COX-2 and iNOS. In addition, luteolin represses activation of NFκB and its upstream molecular factor, Akt (65).

The treatment of ALI mice with the ethanolic extract of *Glycyrrhiza glabra* (200 and 400 mg/kg, p.o., for 4 days) significantly reduced the exudation of protein and the total cell count into the BALF but increased the BALF SOD and CAT activities (66). The alcoholic extract of *Anemarrhena asphodeloides* decreased the inflammatory cells in the BALF and inhibited lung inflammation by its saponin-enriched fraction. The inflammatory markers in the LPS-induced ALI in mice were significantly inhibited by oral administration of timosaponin A-III (67).

The pre-treatment of mice exposed to LPS with crude extract of *Eleusine indica* (400 mg/kg) inhibited the lung neutrophil recruitment 98% dose-dependently. Vitexin (8-C-β-glucopyranosylapigenin) and schaftoside (6-C-β-glucopyranosyl-8-C-α-arabinopyranosylapigenin) isolated from aerial parts (400 μg/kg), inhibited lung neutrophil influx, by 62 and 80%, respectively (68). The pre-treatment with astragalin (25, 50, and 75 mg/kg, p.o., 1 h before LPS challenge), a flavonoid from several medicinal plants, decreased inflammatory responses and improved survival in lethal endotoxemia of a murine model of the LPS-induced ALI. The anti-inflammatory effect of astragalin was correlated with the reduction of IL-1, IL-6, and TNF-α levels produced through the inactivation of NF-κB (69).

The Jojoba oil dry (400 mg, i.t., the air flow rate of 60 L/min for the duration of 7 s) nanoemulsion powders indicated more anti-inflammatory effects on the LPS-induced ALI than dexamethasone with a detrimental effect on the total protein content and down-regulation of TNF-α, IL-1β, IL-6, and NF-κB p65 (116).

Among carvone isomers, pre-treatment with D-carvone (25 and 50 mg/kg, i.g., 1 h before LPS challenge), significantly alleviated the LPS-induced lung injury by diminishing the lung wet/dry ratio and the number of inflammatory cells in the BALF. The serum pro-inflammatory cytokines were remarkably decreased in D-carvone-treated mice. The lung histopathological changes in the LPS-induced lung injury were improved by D-carvone. In addition, the comparable effects of D-carvone with those of dexamethasone were seen (70). Myricetin (10, 20, and 40 mg/kg, 30 min after the LPS challenge), a member of the flavonoid class of polyphenolic compounds, significantly decreased lung inflammation by reduction of the lungs' wet/dry weight ratio, protein levels in the BALF, cytokine levels, and migration of the inflammatory cells. The TLR4, MyD88, and NF-κB expressions were also decreased and the activities of MPO, SOD, GPx, and CAT were increased in the mice exposed to LPS (71).

The administration of petroleum ether fraction of *Viola yedoensis* (2, 4, and 8 mg/kg, p.o.) in the LPS-induced ALI in mice significantly reduced the wet/dry weight ratio of the lung, total inflammatory cells, the activity of MPO, and protein levels in the BALF. The lung morphology improved, the complement deposition was markedly reduced, and the expression of pro-inflammatory cytokines was suppressed in the treated group (72) and pre-treated with rhamnazin (5, 10, and 20 mg/kg, i.p., 2 days before LPS) significantly reduced the inflammatory parameters, improved lung histopathology changes, activated Nrf2 pathway, and attenuated ROS as well as H_2_O_2_, MDA, and hydroxyl ion in the LPS-exposed rats (73). In the LPS-induced lung inflammation and oxidative stress model, the administration of *Nigella sativa* extract (100–400 mg/kg, i.p.) decreased the total and differential WBC counts, oxidative stress, and inflammatory (TGF-β1, IFN-γ, PGE2, and IL-4) markers in the BALF and serum as well as the pathological changes of the lung tissue in the rats (74).

Therefore, the experimental studies showed that pre-treatment with various NP remarkably reduced the inflammatory markers and improved the lung histopathology in the LPS-induced ALI animal models, indicating the therapeutic effect of NP on ALI in the animal models due to their antioxidant and anti-inflammatory properties. The underlying mechanisms of the anti-inflammatory action of NP are inhibition of the Nrf2-mediated antioxidative pathway. Among the biological activities of NP derived from plants anti-inflammatory, antiviral, antitumor, antiallergic, and antioxidant activities can be pointed out. Although many reports have evaluated the effects of these compounds in the experimental models, studies evaluating clinical trials are scarce in the literature. In this section, the effects of different NP on the LPS-induced lung disorders in the experimental models and some possible mechanisms of action were shown. Some experimental data suggest that supplementation with NP may be an effective treatment for patients with the LPS-induced respiratory disorders. The effects of NP on the LPS-induced lung disorders are summarized in Table 4.

**Table 4:** The possible therapeutic effects of medicinal herbs in the LPS-induced lung disorders in experimental studies.

**Study type**	**Study design**	**NP**	**Dose**	**Effects**	**References**
*In vitro*	LPS-stimulated mice RAW264.7 macrophages	*Paulownia tomentosa* methanolic Ext	2.5, 5, 10, 20 and 40 μg/ml, for 24 h	Suppressed IL-6 and TNF-α production	(49)
	LPS-stimulated mice RAW264.7 macrophages	Dehydrodieugenol B from *Nectandra leucantha*	10, 20, 30 and 60 μM	No effect on cell viability Inhibited NO release and IL-1β and IL-6 gene expression	(50)
	LPS-stimulated mice RAW264.7 macrophages	Linalool	40, 80 and 120 μg/mL	↓ TNF-α and IL-6, blocked IκBα protein phosphorylation, p38, c-Jun terminal kinase, and extracellular signal-regulated kinase	(61)
	LPS-stimulated mice RAW264.7 macrophages	Barbaloin from *Aloe vera*	25, 50, or 100 μM	Inhibited IL-1β, IL-6, and TNF-α expression, ROS-mediated PI3K/AKT/NF-κB pathway activation	(51)
	LPS-stimulated mice RAW264.7 macrophages	*Houttuynia cordata* ethanolic Ext	30, 50, 100 and 300 μg/mL	Inhibited NO production	(52)
	LPS-stimulated mice RAW264.7 macrophages	Alpinumisoflavone	1, 5 and 10 μg/mL, for 24 h	↓ NO, TNF-α, IL-6, IL-1β, and ICAM-1 protein expression, IKK and NF-κB phosphorylation, NF-κB nuclei localization, ERK, JNK and p38 phosphorylation, IL-17A and iNOS expression Blocked IκBα phosphorylation and degradation, NLRP3 inflammasome, caspase-1 activation, and IL-1β proteins, ↑ CAT, HO-1, GPx, and SOD, Inhibited intracellular ROS generation	(53)
	LPS-stimulated RAW264.7 macrophages in mice	TADIOS ethanolic Ext	0.5, 1 and 2 mg/mL	↑ Relative luciferase units Suppressed IL-6 and IL-1β, and ROS production *in vivo*	(57)
	LPS-stimulated mice splenocytes and RAW264.7 macrophages	*Curcuma longa* aqueous Ext	0.8-500 μg/mL, for 48 h	↑ NO, IL-12, IL-10, IL-6, IL-2, TNF-α, IFN-γ and MCP-1 in non-stimulated mouse splenocytes and macrophages, Inhibited production of IL-12 and PGE2 in LPS-stimulated cells	(54)
	LPS-stimulated LECL and H460 CCL	*Thymus vulgaris* Hydroalcoholic Ext	0.04-0.60%	↓ NF-κB p52 and NF-κB p65 transcription factors protein, IL-1ß, IL-8 and mucus s Induced necrotic cell death in human H460 lung cancer cell line	(55)
	LPS-stimulated A549 alveolar epithelial cells	Daidzein	100 μM, 15 min after LPS stimulation	Inhibited expressions of TLR4 and MyD88 and the activation of NF-κB	(56)
	LPS-induced ALI in mouse model	TADIOS	1000 mg/kg, orally	↓ Neutrophil infiltration in BALF, inflammatory cell infiltration in lung tissue and thickening of the alveolar wall Activated Nrf2-HO-1 axis	(57)
*In vivo*	LPS-induced ALI in mouse model	Eugenol and Dehydrodieugenol B from *Nectandra leucantha*	30 mg/kg	↓ Lung edema, inflammatory cells, and IL-6 and IL-1 β levels in BALF, iNOS, MMP-9, and TIMP-1, collagen levels and the 8-isoprostane expression in lung tissue, Inhibited phosphorylation of JNK	(50)
	LPS-induced ALI in mouse model	Thymol from *Thymus vulgaris*	30 and 100 mg/kg, i.p.	Improved lung pathological changes ↓ Inflammatory cells influx, TNF-α and IL-6 protein, MDA and MPO l levels in BALF and NF-κB activation in lung ↑ SOD activity,	(58)
	LPS-induced ALI in mouse model	*Pulicaria petiolaris* methanolic Ext	50 and 100 mg/kg, p.o., for 5 days	↓ Lung wet/dry weight (W/D) ratio, total protein and LDH level in BALF, lung histopathological lesions, inflammatory cell infiltration, MDA and ↑ SOD and GSH	(59)
	LPS-induced ALI in mouse model	Cannabidiol from *Cannabis sativa*	0.3, 1.0, 10, 20, 30, 40, and 80 mg/kg, i.p.	↓ Leukocyte and myeloperoxidase activity in lungs, albumin level in BALF, and TNF, IL-6, MCP-1 and MIP-2 production	(60)
	LPS-induced ALI in mouse model	Linalool	25 mg/kg, i.p.	↓ TNF-α and IL-6 production, total WBCs, neutrophils and macrophages in BALF Improved lung histopathologic changes	(61)
	LPS-induced ALI in mouse model	Barbaloin from *Aloe vera*	25, 50, and 100 mg/kg, i.p.	↓ Lung pathological changes, MPO activity and inflammatory neutrophil in lung tissue	(51)
	LPS-induced ALI in mouse model	*Taraxacum mongolicum* Hand.-Mazz aqueous Ext	5 and 10 g/kg, p.o.	↓ Inflammation cells in BALF, protein levels I3K/Akt/mTOR, MPO and inflammatory neutrophil accumulation in lung P ↑ SOD activity	(62)
	LPS-induced ALI in mouse model	*Portulaca oleracea* hydroethanolic Ext	50, 100 and 200 mg/kg, p.o., 1 h before LPS injection	↓ IL-β, IL-6, TNF-α, PGE2, and TGF-β, and increased IL-10 levels, lung wet/dry ratio Improved the level of WBC, MPO, MDA, thiol, SOD and CAT,	(63)
	LPS-induced ALI in mouse model	Xanthohumol from *Humulus lupulus*	10 or 50 mg/kg, i.p.	↓ Lung histopathological changes W/D ratio protein levels, neutrophil infiltration, MDA, MPO, SOD and GSH depletion, ROS, and cytokines levels, iNOS and HMGB1 expression, Txnip/NLRP3 inflammasome and NF-κB signaling pathway activation, t-BHP-stimulated cell apoptosis ↑ Anti-oxidative enzymes expression regulated by Keap1-Nrf2/ARE activation	(64)
	LPS-induced ALI in mouse model	Luteolin	0, 18, 35 and 70 μmol/kg, i.p., for 30 min	↓ Histological changes and lung tissue edema, vascular permeability, TNF-α and IL-6 levels in BALF, and expression of iNOS and COX-2 in lung, NFκB activation upstream molecular factor, Akt	(65)
	LPS-induced ALI in mouse model	*Glycyrrhiza glabra* Ethanolic Ext	200 and 400 mg/kg, p.o., for 4 days	↓ Lung wet/dry weight ratios, lung pathological changes. total cell and protein exudate in BALF, pro-inflammatory mRNA expression Improved SOD activity in BALF,	(66)
	LPS-induced ALI in mouse model	*Anemarrhena asphodeloides*, alcoholic Ext Saponin-enriched fraction, Timosaponin A-III	50 and 200 mg/kg, p.o. 10 and 50 mg/kg 25-50 mg/kg	↓ Total WBCs count, and inflammatory cell infiltration, neutrophil infiltration and macrophages in BALF, IL-1β and IL-6 production in BALF, STAT3 activation, alveolar wall thickness and infiltration of inflammatory cells	(67)
	LPS-induced ALI in mouse model	*Eleusine indica*, Schaftoside and vitexin	4, 40 and 400 mg/kg, i.p. 400 μg/kg, i.p.	Inhibited lung neutrophil influx	(68)
	LPS-induced ALI in mouse model	*Houttuynia cordata* ethanolic Ext Afzelin, hyperoside and Quercitrin	100 and 400 mg/kg, p.o. 100 mg/kg, p.o.	↓ Total cell numbers in BALF ↓ Neutrophils, macrophages and dendritic cells in BALF	(52)
	LPS-induced ALI in mouse model	Astragalin	25, 50 and 75 mg/kg, p.o., 1 h before LPS challenge	Improved animal survival rate, ↓ Lung pathological changes, lung W/D ratio, total protein levelin BALF, total WBC, neutrophils and macrophages in BALF, IκB degradation Down-regulated TNF-α, IL-1β and IL-6 production,	(69)
	LPS-induced ALI in mouse model	D-carvone	25 and 50 mg/kg, i.g., 1 h before LPS challenge	↓ Lung wet/dry ratio, total cells, macrophages, and neutrophils in BALF, TNF-α, IL-1β, and IL-6 in serum	(70)
	LPS-induced ALI in mouse model	Myricetin	10, 20 and 40 mg/kg, 30 min after LPS challenge	↓ Lung wet/dry ratio, protein concentration in BALF, MPO activity, cytokine, and inflammatory cell migration, TLR4, MyD88 and NF-κB expression, ↑ SOD, GPx and CAT levels	(71)
	LPS-induced ALI in mouse model	Petroleum ether fraction of *Viola yedoensis*	2, 4, and 8 mg/kg, p.o.,	↓ Lung wet/dry ratio, total cells, RBC, protein level, and MPO activity in BALF, histopathological damage, expression of TNF-α, IL-1β, and IL-6	(72)
	LPS-induced ALI in rat model	Rhamnazin	5, 10 and 20 mg/kg, i.p., 2 days before LPS	↓ Lung wet/dry ratio, protein level in BALF, LDH and MPO activities, cytokine and oxidative stress, and histopathological damage	(73)
	LPS-induced ALI in rat model	*Nigella sativa* hydroethanolic Ext	100-400 mg/kg, i.p.	↓ Total and differential WBC, MDA, TGF-β1, IFN-γ, PGE2 and IL-4 levels in BALF, and lung pathology ↑ thiol. SOD and CAT levels in BALF and serum	(74)

*NP, natural products; Ext, extract; ALI, acute lung injury; BALF, bronchoalveolar lavage fluid; LECL, lung epithelial cell line; CCL, cancer cell line; COX-2, cyclooxygenase-2; GPx, glutathione peroxidase; GSH, glutathione; HO-1, heme oxygenase-1; i.g., intragastric; i.t., intratracheal; ICAM-1, intercellular adhesion molecule-1; JNK, Jc-Jun-NH2 terminal kinase; LDH, lactate dehydrogenase; MCP-1, monocyte chemoattractant protein-1; MDA, malondialdehyde; MPO, myeloperoxidase; MyD88, myeloid differentiation factor 88; Nrf2, nuclear factor erythroid 2–related factor 2; p.o., per os (by way of the mouth); PGE2, prostaglandin E2; RBC, red blood cells; ROS, reactive oxygen species; SOD, superoxide dismutase; TGF-β, transforming growth factor; TLR4, toll-like receptor 4; TNF, tumor necrosis factor; Taraxacum officinale F.H.Wigg; TADIOS, Dioscorea batatas Decaisne and Schizonepeta tenuifolia*.

*The up arrow (↑) indicates an increase in the variable, and a down arrow (↓) indicates a decrease*.

### Paraquat-Induced Lung Disorders

#### Experimental Studies

The treatment with *Zataria multiflora* (200 and 800 mg/kg) and carvacrol (20 and 80 mg/kg) improved the inhaled PQ-induced systemic oxidative stress and inflammation (75). Also, treatment with carvacrol reduced the WBC (total and differential) count, oxidant biomarkers, and inflammatory cytokines, but increased the antioxidants including CAT and SOD, and anti-inflammatory cytokines in the inhaled PQ-exposed rats similar to the effects of pioglitazone and dexamethasone (76).

The administration of the bark extract of *Bathysa cuspidata* (200 and 400 mg) was shown to protect against PQ-induced acute lung injury and mortality in the rats exposed to PQ as substantiated by the significant decreases in lung edema, septal thickening, alveolar collapse, hemorrhage, cell migration, malondialdehyde, and proteins carbonyl levels (77). In another similar study, salidroside (10 mg/kg), derived from *Rhodiola rosea*, alleviated the PQ-induced lung injury in the rats *via* down-regulation of the TGF-β1 expression (78). In an experimental study, the rats were exposed to a single dose of PQ and treated with pioglitazone (5 mg/kg), pioglitazone plus *Zataria multiflora* extract (200 mg/kg), pioglitazone plus carvacrol (20 mg/kg), and dexamethasone (0.03 mg/kg). The results indicated that the treatment of lung and systemic oxidative stress and inflammation induced by the inhaled PQ in the rats with a combination of pioglitazone plus *Zataria multiflora* or carvacrol showed more effect than the effect of pioglitazone or the plant and carvacrol alone (79).

In the rats exposed to PQ and treated with *Zataria multiflora*, the levels of IL-10, IL-4, TNF-α, and IFN-γ were significantly increased and IL-6, IL-8, and IL-2 levels were decreased (80). The treatment with *Zataria multiflora* (200 and 800 mg/kg) markedly reduced the WBC (total and differential) counts, serum levels of NO_2_, MDA, IL-17, and TNF-α as well as improved the PQ-induced acute lung injuries (81). *Ligustrazine* (30 mg/kg, i.g.), an active substance extracted from the Umbelliferae plant *Ligusticum chuanxiong* (30 mg/kg, i.g.), improved the lipid peroxidation damage, improved the lung injury, and induced the concentrations of NK-κB and iNOS caused by acute poisoning with PQ (82). In the lung injury induced by sub-acute exposure with PQ, treatment with curcumin (30 mg/kg, i.g.) and nano-curcumin-attenuated lung fibrosis may be associated with their antioxidant properties (83).

Diosmin (50 and 100 mg/kg), in a mouse model of PQ-lung injury, showed antioxidant, anti-inflammatory, and antifibrotic effects (84). In a similar study, the PQ-exposed mice treated with the extract of *Rosa canina* fruits (200 and 400 mg/kg) improved the oxidant-antioxidant balance in the lung tissue (85). The treatment of PQ-exposed mice with apigenin (25, 50, and 100 mg/kg) significantly reduced lung injury by inhibition of oxidative stress and inflammation (86).

**Figure 4:**
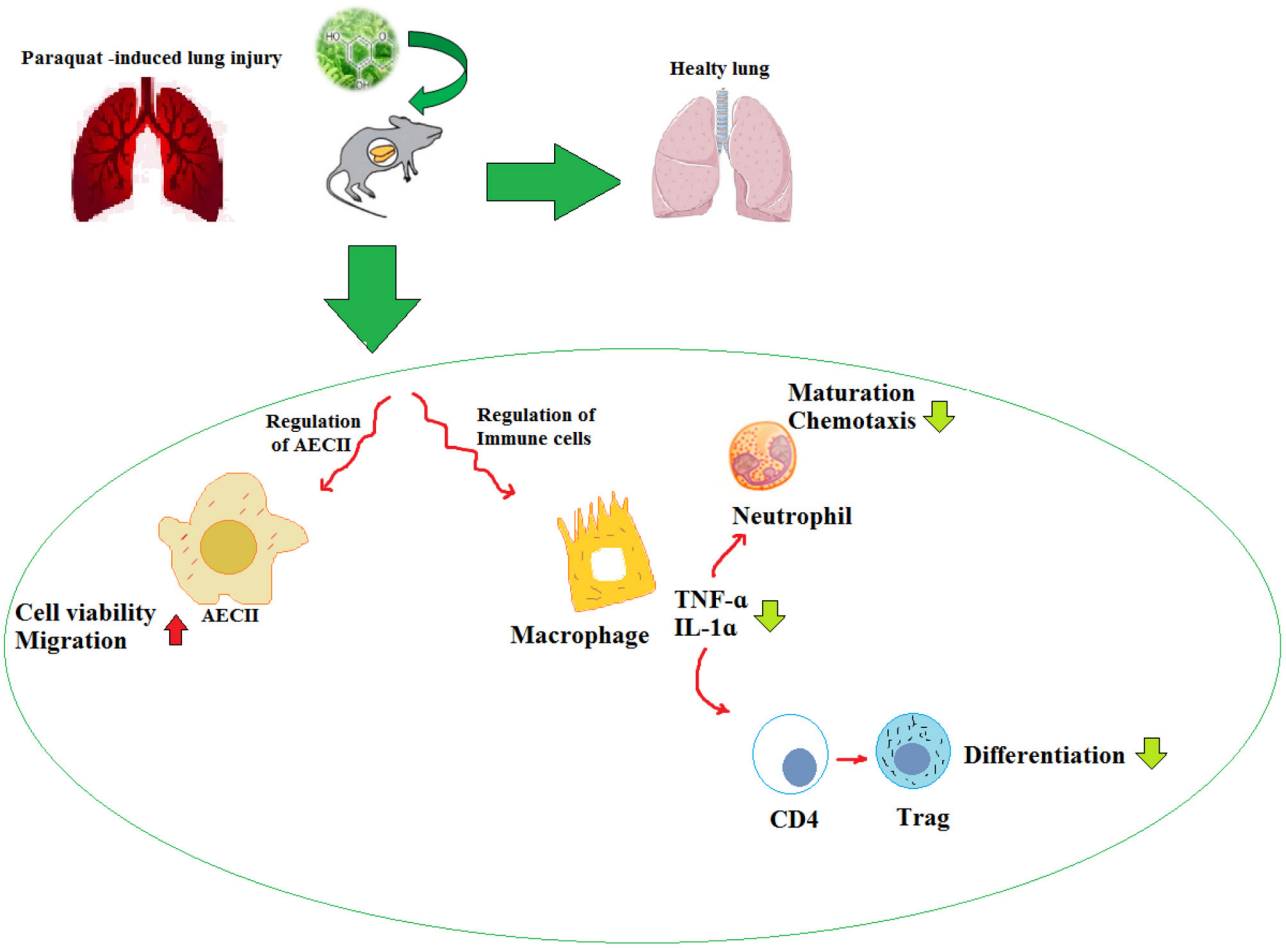
Inhibitory effects of NP on PQ-induced pulmonary fibrosis.

The studies have shown that NP have a variety of medicinal activities including anti-inflammatory, antioxidant, and anticancer. Due to their low water solubility, NP are significantly limited in clinical application. Many potential strategies are expected to be developed to improve their pharmacokinetic values and bioavailability. The experimental evidence supports the causal relationship between oxidative stress and various chronic diseases. Thus, numerous studies are focused on ameliorating the PQ-induced lung injury by decreasing oxidative stress. The experimental studies suggest that NP can combat oxidative stress and reduce the morbidity and mortality associated with PQ-induced lung injury. The effects of NP on PQ-induced lung injury are summarized in Table 5 and Figure 4.

**Table 5:** The possible therapeutic effects of NP in the PQ-induced lung injury.

**Study type**	**Study design**	**NP**	**Dose**	**Effects**	**References**
*In vivo*	PQ-exposed rats	*Zataria multiflora*	200 and 800 mg/kg, i.g. for 16 days	Improved systemic inflammation and oxidative biomarkers	(75)
	PQ-exposed rats	Carvacrol	20 and 80 mg/kg, i.g. for 16 days	↓ Total and differential WBC, MDA, NO2, IL-17 and TNF- α ↑ CAT, SOD activities, IL-10 and INF-γ	(76)
	PQ-exposed rats	*Bathysa cuspidata*	200 and 400 mg/kg, i.g.	↓Lung edema, septal thickening, alveolar collapse, hemorrhage, cell migration, malondialdehyde and proteins carbonyl levels	(77)
	PQ-exposed rats	*Zataria multiflora*, Carvacrol	200 mg/kg 20 mg/kg, i.g. for 16 days	↓ Total and differential WBC, MDA, NO2, IL-17 and TNF- α ↑ CAT, SOD activities, IL-10 and INF-γ	(79)
	PQ-exposed rats	*Zataria multiflora*	200 and 800 mg/kg, i.g. for 16 days	Improved lung inflammation and oxidative stress	(80)
	PQ-exposed rats	*Zataria multiflora*	200 and 800 mg/kg, i.g. for 16 days	↓ Total and differential WBC, IL-17, TNF- α ↑ IL-10, INF-γ	(81)
	PQ-exposed rats	Salidroside	10 mg/kg, i.p.	Suppressed TGF-β1 expression in rat lung injury	(78)
	PQ-exposed rats	ligustrazine	30 mg/kg, i.g.	Improve the lipid peroxidation damage ↓ Lung injury, NK-κB, and iNOS	(82)
	PQ-exposed rats	Curcumin	30 mg/kg, i.g.	↓ Total and differential WBC, IL-17, TNF- α ↑ IL-10, INF-γ	(83)
	PQ-exposed mice	Diosmin	50 and 100 mg/kg, i.p. for 10 or 24 days	Protective effects against PQ-induced lung injury	(84)
	PQ-exposed mice	*Rosa canina*	200 and 400 mg/kg, orally for 14 days	↓ IL-17, TNF- α ↑ IL-10, INF-γ	(85)
	PQ-exposed mice	Apigenin	25, 50 and 100 mg/kg, orally for 7 days	↓ NF-κB, inflammation and oxidative stress	(86)

*NP, natural products; Ext, extract; PQ, paraquat; WBC, white blood cell; MDA, malondialdehyde; NO_2_, nitrogen dioxide; IL-17, interleukin-17; TNF-α, tumor necrosis factor alpha; IFN-γ, interferon gamma; IL-10, interleukin-10; NF-κB, nuclear factor kappa B; iNOS, induced nitric oxide synthase; i.g., intragastrically; i.p., intraperitoneal*.

*The up arrow (↑) indicates an increase in the variable, and a down arrow (↓) indicates a decrease*.

### Sulfur Mustard-Induced Lung Disorders

#### Experimental Studies

The aqueous extract of *Crocus sativus* (225, 450, and 900 μg) decreased DNA damage and MDA but increased the GSH level in the SM-exposed macrophage cells (87).

The effects of antioxidants caffeic acid (CA) (250 μM) and quercetin (100 μM) on normal human epithelial keratinocytes (NHEKs) treated with SM (200 μM) showed their protective effects on the cytotoxicity induced by SM. Also, CA improved cell viability at concentrations > 250 μM dose-dependently. In addition, the treatment with CA and quercetin decreased the phosphorylation of p38 and p53 but increased the phosphorylation of JNK 1/2 induced by SM. Furthermore, CA and quercetin reduced the expression levels of cyclooxygenase 2 (COX-2), inducible NO synthase (iNOS), and the induction of oxidative stress irrespective of the p38 and lipoxygenase pathway (88).

In female mice exposed to SM, ethanolic extract of *Hippophae rhamnoides* L. leaf (HL-EOH), water and ethanolic extract of *Hippophae rhamnoides* fruit (HF-W and HF-EOH), and *H. rhamnoides* flavone from fruit (HR-flavone) significantly protected the lethal effect of SM. Treatment with HL-EOH and HR-flavone markedly protected the bodyweight loss, levels of GSH, oxidized glutathione (GSSG), and MDA. The LD_50_ of all extracts was more than 5 g/kg indicating their non-toxic property (89).

The *Nigella sativa* extract (0.08 g/day) in drinking water markedly decreased airway responsiveness to methacholine and WBC count in the guinea pigs exposed to SM (90). Similarly, the *Nigella sativa* extract at the above dose for 2 weeks also remarkably reduced airway responsiveness, neutrophil, eosinophil, lymphocyte, and monocyte percentage in the SM-exposed guinea pigs and the effects of *Nigella sativa* were similar to the onset of dexamethasone (5 mg/kg, i.p.) (91). The therapeutic effects of *Salvia miltiorrhiza* and *Anemarrhena asphodeloides* mixture (MSTF) (30, 60, 120 mg/kg) after exposure of the rats to SM (3.5 mg/kg, s.c.) significantly enhanced the survival and diminished the SM-induced morphological changes in the liver, small intestine, and testis tissues. The administration of MSTF (60 and 120 mg/kg) markedly increased the GSH level and prevented the differential expression of genes in the SM-exposed rats (92).

#### Clinical Studies

In a case-control study, the treatment of patients with lung disorder due to SM exposure (n=20) with boiled extract of *Nigella sativa* (0.375 ml/kg) significantly improved the PFT values, and chest wheeze 30 and 60 days after treatment compared to the placebo-treated group (n=20) and also compared to the beginning of the study (93). The treatment effects of the *Avena sativa* plant (0.1% cream twice a day for 4 weeks) on chronic pruritus in the SM-exposed patients in a double-blind clinical trial were studied. A total of seventy-five patients were divided into 3 groups including *Avena sativa* ointment, placebo, and betamethasone groups. At the end of the study period, the pruritus severity was significantly reduced in groups A and B compared to group C. The *Avena sativa* ointment treatment also improved the quality of life and quality of sleep in the patients (94).

In a randomized clinical study, the treatment of patients with lung disorder due to SM exposure with a syrup made from *Zataria multiflora* (5 and 10 mg/kg) for 2 months) reduced the WBCs (total and different) and oxidant biomarker but increased thiol, SOD, and CAT activities, and increased the PFT values (20). In a similar study, the serum levels of inflammatory mediators were reduced but the PFT values were increased due to a 2-month treatment with *Zataria multiflora* in the patients with lung disorders due to SM exposure (95). The treatment with *Zataria multiflora* extract in these patients also diminished cytokines, and respiratory symptoms, but increased some PFT values (96).

The 2-month treatment of the patients with lung disorder for a long time (27–30 years) exposing to SM with carvacrol (1.2 mg/kg) significantly enhanced the CAT and SOD activities, thiol level, and PEF values, but, declined the MDA level, total WBC and neutrophil count (21). The 2-month treatment with carvacrol in similar patients also remarkably reduced the respiratory symptoms and serum levels of IL-2, IL-4, IL-6, IL-8, EGF, and VEGF, but incremented the IFN-γ and IL-10 levels in the serum. In addition, the carvacrol treatment increased MEF25, 50, and 75 (maximum expiratory flow at 25, 50, and 75% of vital capacity) and MMEF (maximum mid-expiratory flow) values after 2 months of treatment (97, 98).

In the patients exposed to SM with chronic pruritic skin lesions (*n* = 96) treated by curcumin (1 g/d) or placebo for 4 weeks, the serum levels of high-sensitivity C-reactive protein (hs-CRP) and IL-8 were reduced in both groups. However, a higher effect of curcumin was observed compared to the placebo group and only treatment with curcumin reduced the calcitonin gene-related peptide (CGRP) level. In addition, in the curcumin group, IL-8 was correlated with the dermatology life quality index (DLQI) change (99). The treatment with curcumin in SM-induced chronic pruritus also improved the antioxidant status, quality of life (QoL), and pruritus (100).

The treatment of the patients with pulmonary complications induced by SM, with curcuminoids (500 mg) for 4 weeks, increased FEV1/FVC compared to placebo-treated patients. The inflammatory mediators (IL-6, IL-8, TGFβ, TNFα, hs-CRP, substance P, CGRP, and MCP-1 also improved remarkably greater than the placebo group. Therefore, in patients with SM-induced chronic pulmonary disorders, short-term curcuminoids treatment reduced lung and systemic inflammation (101).

In the other study, the SM-exposed patients were treated with standard drugs plus curcuminoids and piperine (1,500 and 15 mg/day, respectively) or placebo for 4 weeks. The serum level of GSH was increased but that of MDA decreased and the health-related quality of life (HRQoL) was significantly improved at the end of the study in both groups. However, GSH, MDA, and the HRQoL changes in the curcuminoids-piperine-treated group were markedly greater than the placebo group (102).

**Figure 5:**
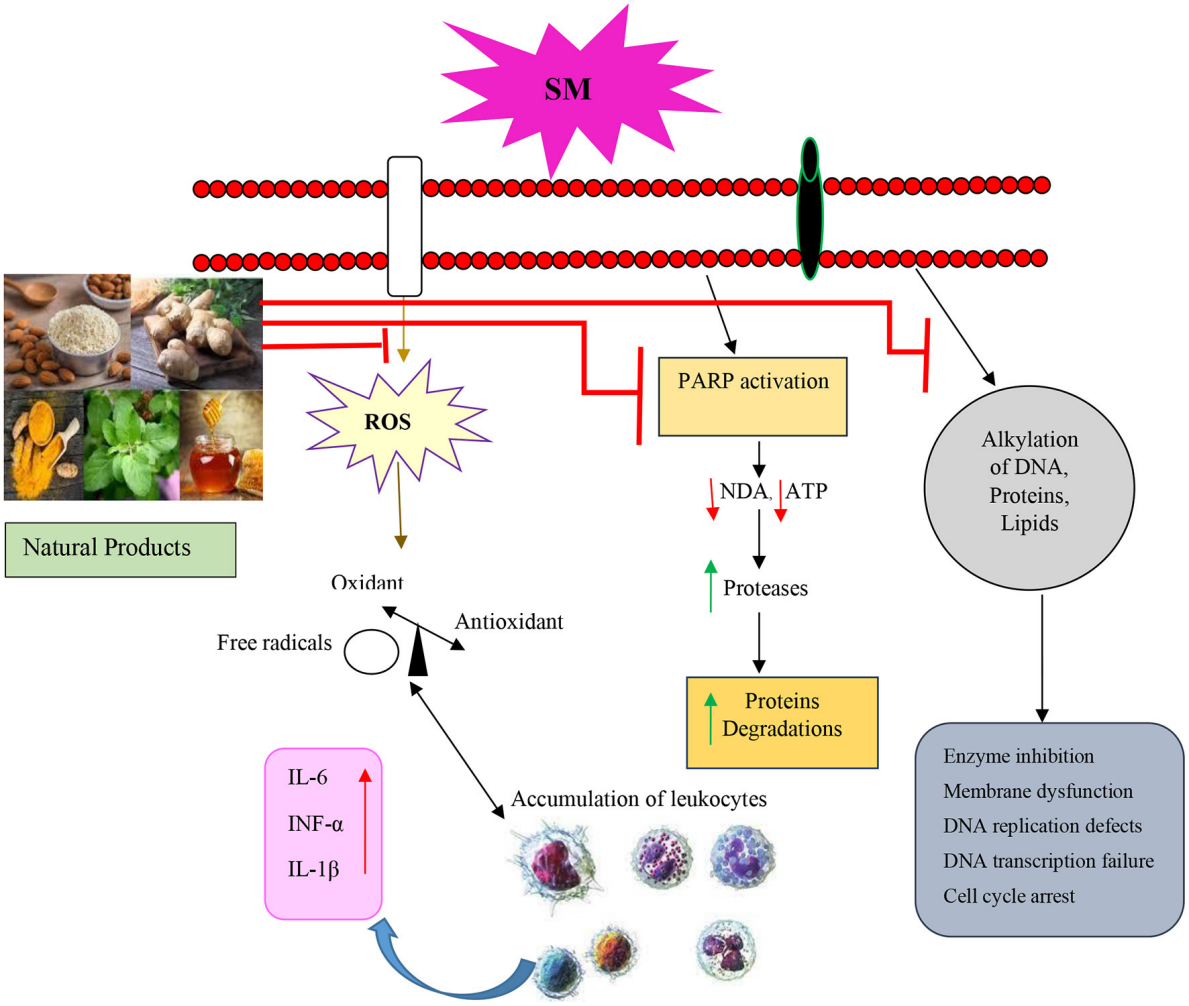
Molecular mechanism of SM-induced toxicity of the relevant studies. SM, Sulfur mustard; ROS, Reactive oxygen species; PARP, Poly (ADP-ribose) polymerase; IL, Interleukin; TNFα, Tumor necrosis factor-α.

The above studies showed the protective effect of NP on the cell viability, inflammation, and pathological changes in the experimental studies that were exposed to SM. The clinical studies also indicated that NP improve the quality of life, PFT values, respiratory symptoms inflammatory mediators, and oxidative stress markers of SM-induced lung disorder. The therapeutic effects of NP in SM-induced lung injury are summarized in Table 6 and Figure 5. The molecular mechanism of SM-induced toxicity is shown in Figure 5.

**Table 6:** The possible therapeutic effects of medicinal plants and their derivatives in the SM-induced lung injuries.

**Study type**	**Study design**	**NP**	**Doses**	**Effects**	**References**
*In vivo*	Macrophage	*Crocus sativus*	225, 450 and 900 μg	↓ DNA damage and MDA ↑ GSH level in macrophage cells exposed to SM.	(87)
	SM-exposed NHEKs	Caffeic acid and quercetin	250 and 100 μM	↓ p38 and p53 phosphorylation, expression levels of COX2 and iNOS and oxidative stress ↑ JNK 1/2 phosphorylation	(88)
	SM-exposed Swiss female mice	*Hippophae rhamnoides* ethanolic Ext	1g/kg; 3 doses; p.o	Protected the body weight loss ↑ GSH, and GSSG levels ↓ MDA w	(89)
	SM-exposed guinea pigs	*Nigella sativa*	0.08 g/day	↓ TR to methacholine, total and differential WBC count	(90)
	SM-exposed guinea pigs	*Nigella sativa*	0.08 g/day	↑ TR and lung in?ammation similar to the effect of dexamethasone	(91)
	SM-exposed Sprague Dawley rats	*Salvia miltiorrhiza Anemarrhena asphodeloides*	30, 60, 120 mg/kg	↑ Survival levels of rats ↓ The SM-induced morphological changes in the testis, small intestine and liver tissues	(92)
Clin	SM-exposed patients	*Nigella sativa* aqueous Ext	0.375 mL/kg	Improved chest wheeze, PFT values	(93)
	SM-exposed patients	*Avena sativa*	%5 w/w	Improved disease severity, quality of life and quality of sleep	(94)
	SM-exposed patients	*Zataria multiflora*	5 and 10 mg/kg	↓ Total and different WBC, MDA ↑ Thiol, CAT, SOD, FVC and PEF	(20)
	SM-exposed patients	*Zataria multiflora*	-	Improved serum levels of various cytokines, chemokine's and PFT values	(95, 96)
	SM-exposed patients	Carvacrol	-	↑ Thiol level, CAT and SOD activity and PE ↓ Total WBC and MDA	(21)
	SM-exposed patients	Carvacrol	1.2 mg/kg/day	↓ Respiratory symptoms, EGF, VEGF, IL-8, IL-2, IL-6 and IL-4 in the serum, ↑ Serum levels of IL-10 and IFN-γ and PFT values	(97, 98)
	SM-exposed patients	Curcumin	-	↓ hs-CRP, CGRP and IL-8 serum levels and DLQI	(99)
	SM-exposed patients	Curcumin	1 g/day	Improved HQoL, pruritus, and antioxidant status	(100)
	SM-exposed patients	Curcuminoids	500 mg	Improved FEV1/FVC, IL-6, IL-8, TGFβ, TNFα, hs-CRP, SP, CGRP and MCP-1. Also	(101)
	SM-exposed patients	Curcuminoids + piperine	1500 and 15 mg/day	Improved HRQoL, GSH, MDA	(102)

*NP, natural products; Clin, clinical; Ext, extract; SM, sulfur mustard; NM, nitrogen mustard; TR, tracheal responsiveness; WBC, white blood cells; GSH, glutathione; VEGF, vascular endothelial growth factor; NO, nitric oxide; MDA, malondialdehyde; GSH, glutathione; GSSG, oxidized glutalthione; SOD, superoxide dismutase; CAT, catalase; IL, Interleukin; IFNγ, interferon γ; TNFα, tumor necrosis factor-α; COX-2, cyclooxygenase-2; iNOS, inducible nitric oxide synthase; SIL-BS, silibinin-bis-succinat; PFT, pulmonary function test; FEV1, volume in one second; MMEF, maximal mid expiratory Low; FVC, forced volume capacity; hs-CRP, high-sensitivity C-reactive protein; CGRP, calcitonin gene-related peptide; VEGF, vascular endothelial growth factor; EGF, epidermal growth factor; DLQI, dermatology life quality index; HRQoL, health-related quality of life; SP, substance P*.

*The up arrow (↑) indicates an increase in the variable, and a down arrow (↓) indicates a decrease*.

### Other Noxious Agents-Induced Lung Disorders

#### Experimental Studies

The pre-treatment with *Origanum vulgare* extract (50, 100, 200, and 400 mg/kg) protected the lung tissues from cyclophosphamide (CP)-induced pulmonary damage and suggested a role for oxidative stress in the pathogenesis of the lung disease induced by CP (103). In the rats challenged with methotrexate (MTX) (20 mg/kg) and treated with alpha-lipoic acid (ALA) after MTX administration, the levels of IL-1β, MDA, GSH, TNF-α, MPO, and sodium potassium-adenosine triphosphatase (Na+/K+ATPase) were improved due to the ALA treatment (104).

In a rat model of amiodarone-induced lung insult, the serum levels of TGF-β1 and TNF-α markedly increased. The treatment with grape seed extract (150 mg/kg) ameliorated oxidative and fibrotic damages in the lung of the amiodarone-treated rats (105). The aqueous extract of caffeic acid phenethyl ester (5 and 10 μmol /day) significantly attenuated the acute lung injury induced by amiodarone (7.5 UI/kg). The activities of myeloperoxidase and SOD enzymes were significantly decreased in the group which was treated with caffeic acid phenethyl ester (106). In the amiodarone-induced lung toxicity, two phenolic acids, ferulic acid and gallic acid, showed a protective effect on the inflammatory biomarkers and oxidative stress (107).

A protective effect of *A. melanocarpa* fruit juice against amiodarone-induced pulmonary toxicity was shown by the reduction of amiodarone-induced direct toxic damage signs, oxidative stress, and fibrosis (108). Treatment with grape seed and Ginkgo biloba (100 mg/kg) ameliorated the histopathological structure, increased the contents of glycogen, and improved the ultrastructure alternations of the lung tissue in the rats exposed to a single dose of amiodarone (40 mg/kg). Grape seed was markedly more effective than Ginkgo biloba in protecting the rats against amiodarone (109). The therapeutic effects of NP in the other noxious agents-induced lung injury are summarized in Table 7 and Figure 3.

**Table 7:** The effect of other noxious agents-induced lung disorders.

**Study type**	**Study design**	**NP**	**Dose**	**Effects**	**References**
*In vivo*	CP-exposed mice	*Origanum vulgare*	50, 100, 200 and 400 mg/kg, i.p. for 7 days	↓ IL-6, IL-8, mRNA, protein expression and NF-κB activity	(103)
	MTX-exposed rats	Alpha-lipoic acid	60 mg/kg, i.p. for 16 days	↓ IL-2, IL-4, IL-6, IL-8 ↑ IL-10, IFN-γ, IFN-γ/IL-4 ratio	(104)
	AM-exposed rats	Grape seed Ext	150 mg/kg, i.p for 14 days	↓ Serum levels of IL-4, IL-17A, IFN γ ↑ TGFβ	(105)
	AM-exposed rats	Caffeic acid phenethyl ester	5 and 10 μmol /day, i.p. for 3 weeks	↓ MDA level and the activity of myeloperoxidase ↑ SOD	(106)
	AM-exposed rats	ferulic acid, gallic acid	200 and 100 mg/kg, i.g., for 6 weeks	Improved inflammatory biomarkers and oxidative stress	(107)
	AM-exposed rats	*A. melanocarpa* fruit juice	5 and 10 mL/kg, orally for 10 days	↓ Oxidative stress, inflammation, and fibrosis	(108)
	AM-exposed rats	Grape seed and ginkgo biloba	100 mg/kg, i.g. for 8 weeks	↓ Antioxidant's and histopathological structure ↑ The contents of glycogen	(109)

*NP, natural products; Ext, extract; CP, cyclophosphamide; MTX, methotrexate; AM, Amiodarone; MDA, Malondialdehyde; SOD, superoxide dismutase; IL, Interleukin; NF-κB, Nuclear factor kappa B; IFN-γ, Interferon gamma; TGFβ, Transforming growth factor beta; i.g., intragastrically; i.p., intraperitoneal*.

*The up arrow (↑) indicates an increase in the variable, and a down arrow (↓) indicates a decrease*.

## Discussion

The induction of various lung disorders due to exposure to NA of the general environment or in the workplace was shown, both in animal and human studies. Among the most important NA, exposure to BLM, Cd, environmental dust, LPS, PQ, SM, and amiodarone can cause lung diseases. Exposure to these NA usually leads to PF and COPD but it also can induce various other lung disorders such as emphysema. The induction of lung disorders due to exposure to NA is accomplished with lung pathological changes, wet/dry lung weight disturbance, oxidative stress in the lung, lung inflammation indicated by increased inflammatory mediators, and immune dysregulation indicated by the changes in the cytokine levels and other immune markers in the BALF or lung tissues.

The results of this review study showed the pharmacological and therapeutic effects of different NP including medicinal plants and their derivatives on lung disorders both in the experimental and clinical studies. The experimental studies indicated the effects of different medicinal plants including *Aloe vera, Anemarrhena asphodeloides, Avena sativa, Crocus sativus, Curcuma longa, Dioscorea batatas, Glycyrrhiza glabra, Gentiana veitchiorum, Gentiopicroside, Houttuynia cordata, Hibiscus sabdariffa, Hochu-ekki-to, Hippophae rhamnoides, Juglans regia, Melanocarpa fruit juice, Mikania glomerata, Mikania laevigata, Moringa oleifera, Myrtus communis L., Lamiaceae, Myrtle, Mosla scabra leaves, Nectandra leucantha, Nigella sativa, Origanum vulgare L, Pulicaria petiolaris, Paulownia tomentosa, Pomegranate seed oil, Raphanus sativus L. var niger, Rosa canina, Schizonepeta tenuifolia,Thymus vulgaris, Taraxacum mongolicum, Tribulus Terrestris, Telfairia occidentalis, Taraxacum officinale, TADIOS, Xuebijing, Viola yedoensis, Zataria multiflora, Zingiber officinale, Yin-Chiao-San*, and their derivatives on the lung injury induced by NA. The treatment with NP in NA-induced lung disorders ameliorated all lung changes induced by NA such as lung pathological changes, lung oxidative stress, lung inflammation, and immune dysregulation. In clinical studies, the effects of medicinal plants and their derivatives such as *Avena sativa, Curcuma longa, Nigella sativa*, and *Zataria multiflora* on SM-induced lung disorders were shown by reducing respiratory symptoms, oxidative stress markers, inflammatory mediators, and cytokine levels as well as increasing PFT.

The results of this review study showed the possible therapeutic effects of various NP on NA-induced lung disorders by amelioration of various features of lung injury. However, further clinical studies, especially on the effect of NP on lung diseases induced by BLM, Cd, environmental dust, LPS, PQ, and other noxious agents are needed to support the therapeutic effect on NP on NA-induced lung disorders for clinical practice purposes.

## Author Contributions

SS, SB, and MK prepared the first draft of the manuscript and helped in the revision of the final version of the manuscript. MB designed the study, critically edited, and revised the manuscript. All authors contributed to the article and approved the submitted version.

## Conflict of Interest

The authors declare that the research was conducted in the absence of any commercial or financial relationships that could be construed as a potential conflict of interest.

## Publisher's Note

All claims expressed in this article are solely those of the authors and do not necessarily represent those of their affiliated organizations, or those of the publisher, the editors and the reviewers. Any product that may be evaluated in this article, or claim that may be made by its manufacturer, is not guaranteed or endorsed by the publisher.

## Glossary

Ach: acetylcholineAHR: hyperresponsivenessBALF: Broncho-alveolar lavage fluidCAT: catalaseCGRP: calcitonin gene-related peptideCOPD: Chronic obstructive pulmonary diseaseCW: Chemical warfareDLQI: Dermatology Life Quality IndexDOX: DoxycyclineEPO: peroxidaseFDA: Food and Drug AdministrationFEV1: Forced expiratory volume in the first secondFVC: Forced vital capacityGSH: glutathioneHDAC: Histone deacetylase complexhPBMCs: human peripheral blood mononuclear cellsHRQoL: health-related quality of lifehs-CRP: high-sensitivity C-reactive proteinIFNγ: Interferon-gammaIg: immunoglobulinIL: InterleukiniNOS: induced nitric oxide synthaseIPF: idiopathic pulmonary fibrosisLPS: LipopolysaccharideLTC_4_: leukotriene C_4_MCHC: mean corpuscular hemoglobin concentrationMCP-1: monocyte chemotactic protein-1MCV: mean corpuscular volume

MDA: malondialdehydemMCP-1: mouse mast cell protease-1MMEF: maximum mid-expiratory flowMMPs: matrix metalloproteinaseNAC: N-AcetylcysteineNFkB: nuclear transcription factorNK cells: natural killer cellsNO: oxide productionOVA: OvalbuminPaCO_2_: arterial blood carbon dioxidePE: pulmonary embolismPEF: peak expiratory flowPF: pulmonary fibrosisPFT: Pulmonary function testsPQ: paraquatQoL: quality of lifeROS: Reactive oxygen speciesSaO_2_: arterial blood oxygenation saturationSIL-BS: silibinin-bis-succinatSM: Sulfur mustardSOD: superoxide dismutaseTAT: Thrombin anti-thrombinTFPI: Tissue factor pathway inhibitorTGF-β1: transforming growth factor beta1TNFα: Tumor necrosis factor-αtPA: Tissue plasminogen activatorTR: tracheal responsivenessTSM: tracheal smooth muscleUV: ultravioletWBC: White blood cells.
